# Interim data monitoring in cluster randomised trials: Practical issues and a case study

**DOI:** 10.1177/17407745211024751

**Published:** 2021-06-22

**Authors:** K Hemming, J Martin, I Gallos, A Coomarasamy, L Middleton

**Affiliations:** 1 Institute of Applied Health Research, University of Birmingham, Birmingham, UK; 2 University of Birmingham, Birmingham, UK

**Keywords:** Data monitoring, cluster randomised trials, selection bias

## Abstract

**Background:**

There is an abundance of guidance for the interim monitoring of individually randomised trials. While methodological literature exists on how to extend these methods to cluster randomised trials, there is little guidance on practical implementation. Cluster trials have many features which make their monitoring needs different. We outline the methodological and practical challenges of interim monitoring of cluster trials; and apply these considerations to a case study.

**Case study:**

The E-MOTIVE study is an 80-cluster randomised trial of a bundle of interventions to treat postpartum haemorrhage. The proposed data monitoring plan includes (1) monitor sample size assumptions, (2) monitor for evidence of selection bias, and (3) an interim assessment of the primary outcome, as well as monitoring data completeness. The timing of the sample size monitoring is chosen with both consideration of statistical precision and to allow time to recruit more clusters. Monitoring for selection bias involves comparing individual-level characteristics and numbers recruited between study arms to identify any post-randomisation participant identification bias. An interim analysis of outcomes presented with 99.9% confidence intervals using the Haybittle–Peto approach should mitigate any concern regarding the inflation of type-I error. The pragmatic nature of the trial means monitoring for adherence is not relevant, as it is built into a process evaluation.

**Conclusions:**

The interim analyses of cluster trials have a number of important differences to monitoring individually randomised trials. In cluster trials, there will often be a greater need to monitor nuisance parameters, yet there will often be considerable uncertainty in their estimation. This means the utility of sample size re-estimation can be questionable particularly when there are practical or funding difficulties associated with making any changes to planned sample sizes. Perhaps most importantly interim monitoring has the potential to identify selection bias, particularly in trials with post-randomisation identification or recruitment. Finally, the pragmatic nature of cluster trials might mean that the utility of methods to allow for interim monitoring of outcomes based on statistical testing, or monitoring for adherence to study interventions, are less relevant. Our intention is to facilitate the planning of future cluster randomised trials and to promote discussion and debate to improve monitoring of these studies.

## Background

Cluster randomised trials (CRTs) are a firmly established alternative to individually randomised trials.^1^ While there is a growing body of methodological work on how to monitor individually randomised trials^2–4^ and technical literature on how these methods can be extended to CRTs,^5–9^ practical resources for monitoring cluster trials is limited.^3^ Cluster trials have many different features to individually randomised trials. Many cluster trials evaluate low-risk interventions, so monitoring for harm will not always be appropriate. Sample size estimation in cluster trials rests on a greater number of nuisance parameters and the uncertainty of these parameters even at any sample size re-estimation is likely to be high.^10^ Cluster trials also often recruit participants post-randomisation and unblinded to the treatment allocation, and this might require monitoring for evidence of possible selection bias. In practice, monitoring also needs to be actionable, yet logistical constraints might mean theoretical actions (such as increasing the sample size, or recruitment blind to treatment allocation) are not always feasible. Furthermore, CRTs are often pragmatic and evaluate low-risk interventions, so monitoring for harm or monitoring adherence to interventions, might not always be appropriate.^3^

In this article, we outline interim monitoring considerations that are specific and most salient for CRTs. Broadly the aspects of interim monitoring we consider in turn are as follows: the review of sample size assumptions, monitoring for selection bias, monitoring for any signal of harm or benefit, and monitoring adherence to interventions (protocol violations). For each of these, we additionally consider the barriers and practical issues that need to be considered before implementation of any monitoring plan. We then apply these considerations to a case study, the E-MOTIVE trial. The E-MOTIVE trial is a large CRT of an intervention to promote early detection of postpartum haemorrhage evaluated across 80 health facilities in four countries. Our intent is to facilitate the planning of future CRTs and to promote discussion and debate to improve monitoring of these studies.

### Interim monitoring of parameters that inform the sample size calculation

Estimation of sample size in CRTs is highly dependent on *nuisance* parameters which measure the degree of clustering, as well as those typically needed to inform the sample size under individual randomisation. Sometimes, data from a closely related source are available, but in many cases, trials are started with limited knowledge of these parameters. Interim assessments have the potential to inform if the study is likely to be able to detect target effect sizes with reasonable power, and if not, to contribute to a decision on whether the sample size should be increased or (at the other extreme, in exceptional circumstances) whether the study should stop early. In cluster trials with very large sample sizes, sample size re-estimation might be able to inform the utility of recruiting additional participants in clusters (i.e. increasing cluster sizes).^11^ This is formally referred to as sample size re-estimation, a procedure under which the study size is revised upwards or downwards on the basis of interim assessments of nuisance parameters^5,6^ and is often viewed as part of an internal pilot study.^10,12^ Of note, sample size re-estimation might also include estimation of the treatment effect, which we consider under interim monitoring of outcomes. There are several important practical considerations when implementing sample size re-estimation.

*First*, sample sizes needed at interim points to estimate these nuisance parameters with sufficient precision can be high in cluster trials.^10^ Any estimates of confidence intervals around nuisance parameters should allow for the clustered nature of the study. This means not only appropriate consideration of clustering, but this might also require the use of small sample corrections in trials with less than about 40 clusters.^13^ In practice, this means there will often be considerable uncertainty around the values of these nuisance parameters at any sample size re-estimation.

*Second*, sample size re-estimation, by updating nuisance parameters, uses data that will subsequently be used to determine treatment effectiveness (despite not estimating the treatment effect). Because of this, in individually randomised trials, it is established that sample size re-estimation could lead to some small inflation of the type-1 errors,^14^ and this has also been observed in sample size re-estimation in cluster trials.^5,6^ The greater the number of sample size re-estimations or number of nuisance parameters, the higher this inflation. In individually randomised trials, conducting sample size re-estimation without knowledge of treatment indicators (known perhaps confusingly as ‘blinded sample size re-estimation’) reduces this risk,^14–16^ and this is achieved using pooled data to estimate nuisance parameters^15^ and methods exist to extend this to some cluster trial designs.^9^

*Finally*, sample size re-estimation procedures assume that sample sizes will be increased or decreased according to the revised estimates of nuisance parameters. Yet, the nature of cluster randomisation means these nuisance parameters will often be re-estimated with high uncertainty (i.e. wide confidence intervals); and sometimes it might not even be possible to estimate all nuisance parameters (a point to which we return in the case study). Furthermore, in many cluster trials, there will be a real limit on upper bounds for sample size (either because of funding or logistical constraints).^6^ Monitoring these parameters might therefore not always be actionable. Moreover, where a sample size re-estimation indicates the study is overpowered, investigators may be very reluctant to reduce the sample size, particularly where the target difference might be overoptimistic. Where a trial looks to be under-powered investigators might be equally reluctant about stopping the trial.

### Monitoring for selection bias

Cluster trials are at risk of identification and recruitment bias which occur when recruiting (or identifying) individual participants after randomisation of clusters (collectively referred to as selection bias).^17–21^ The risk of this bias is particularly high when the intervention is not blinded and where there is direct patient recruitment. In one trial, this resulted in baseline differences between intervention and control arms, of a magnitude equivalent to the target effect size.^22^ Reviews have identified that between 20% and 40% of cluster trials might exhibit this sort of bias.^19,20,23^ Monitoring cluster trials for signs of identification or recruitment bias, manifested by differences in those recruited under intervention and control arms, could plausibly be used as a means for identifying biases before they undermine the validity of the trial.

The first consideration is whether such monitoring of imbalance should be conducted. Baseline ‘testing’ of differences across arms is known to result in multiplicity issues and is not recommended in individually randomised trials. Yet, some have argued that baseline testing in cluster trials might be appropriate because of the deviation from a random assignment at the level of the individual.^24^ To minimise issues around multiplicity, we suggest monitoring for imbalance on pre-specified individual-level characteristics known to be prognostic of the outcome; and this should only be operationalised for trials in which either identification or recruitment occurs after randomisation and the intervention is unblinded. The number and timings should be pre-specified and kept to a minimum and chosen with consideration of the degree of precision that will be available.

The second consideration is how any imbalance should be identified. Statistical criteria used for identifying imbalance could be either using a global test of balance across all prognostic factors,^25^ separate and independent tests, or as estimates of differences along with confidence intervals (all allowing for the clustered nature of the trial). Statistically nonsignificant differences can still be consistent with biases. Likewise, particularly in large trials, statistically significant differences might not indicate differences of any important magnitude. Therefore, trying to identify trials that exhibit selection bias cannot rest solely on statistical significance and must consider sizes of differences. Pre-specification of clinically important differences is one possible option. We suggest that decisions on how to interpret evidence of selection bias should be by consensus (e.g. between members of the data monitoring committee and trial steering committee). Yet, whether selection biases can be reliably assessed at interim monitoring is yet to be generally determined, although there are anecdotal examples of it having been used successfully.^20^

Finally, if implemented and detected, careful consideration is needed around the actions to be taken. In some situations, any clearly identified imbalance could be followed by changes to strategies of identification and recruitment.^20^ In rare situations of major imbalance, consideration to stopping the trial might be warranted. In other less-extreme cases, systematic differences between intervention conditions, might indicate changes to the analysis plan, for example, by changing any pre-specified unadjusted (other than adjustment for cluster-level covariates used in the randomisation) primary analysis to an adjusted (for individual-level covariates) primary analysis. Finally, showing the absence of substantive imbalance can reinforce any primary unadjusted analysis.

Furthermore, where there is a concern that selection bias might be a problem (i.e. in trials with unblinded post-randomisation participant recruitment), a precautionary approach might consider a pre-specified adjusted analysis as the primary analysis (at the trial end). As others have recommended, any plan for individual-level covariate adjustment should be pre-specified and not based on perceived differences, but rather on covariates which have known prognostic importance. For binary outcomes with low prevalence, this might mean the use of propensity scores, which have been shown to both improve power and reduce the risk of model non-convergence with covariate adjustment.^26^

### Monitoring harms and interim assessments of primary outcome

Interim assessments of individually randomised trials are often concerned with identification of harm and assessments of primary outcomes, as well as fulfilling regulatory requirements and require careful consideration to ensure type-1 errors are not inflated.^2,27–29^ Cluster trials often have less emphasis on harm because they mostly evaluate low-risk interventions.^3^ For those cluster trials that evaluate interventions at greater risk of causing harm, similar principals as those used in individually randomised trials are likely to hold. There may also be interest in assessing primary outcomes at interim assessments, perhaps to identify early signals of benefit. Again, the first consideration here is whether the monitoring is required. Whether interim assessments of outcomes and harm are required will be dependent on the context and the nature of the intervention. Overzealous use of interim monitoring of outcomes is likely to be as unhelpful as no monitoring and so we argue that careful consideration is needed.

When implemented in cluster trials, any interim analysis of outcomes must take steps to ensure type-1 errors are not inflated.^7,8^ In pragmatic trials where the focus tends to be more aligned with effects supported by confidence intervals, these methods can be extended to confidence interval estimation^7^ similarly to how they are extended to confidence interval approaches in individually randomised trials.^30^ Simple to implement methods, which allow for multiplicity using a very stringent p value (such as p < 0.001) at the interim assessment, known as the Haybittle–Peto approach, preserve the type-1 error while not changing the interpretation at the final assessment, although this method has not been validated in cluster trials.

Alternative approaches, known as group-sequential methods, allow for sequential data analysis in accordance with pre-defined stopping rules but without fixing the sample size in advance. These methods combine sample size re-estimation with interim assessments of outcomes. Group-sequential methods have been extensively studied under individual randomization^31–33^ and have been extended to cluster trials.^7,8,34^ A related approach, known as conditional power methods, sequentially calculate the conditional probability of a no effect at the end of the trial,^35^ and again these methods have been extended to cluster trials.^36^

Finally, when implemented, interim analyses require transparency over blinding of treatment conditions. In some cluster trials, monitoring blinded to treatment allocation will not be possible, for example where there are large differences in sample sizes across arms (for example, in unbalanced designs like stepped-wedge trials). Others have argued against blinded monitoring for harm or efficacy irrespective of whether it is feasible.^28^

### Monitoring adherence to assigned interventions

Individually randomised trials might monitor adherence with assigned intervention allocation.^2^ In explanatory trials, where the objective is to provide evidence of efficacy under ideal and controlled circumstances, monitoring adherence can be useful; identified lack of adherence can be actioned upon with steps to improve compliance. However, in pragmatic trials, where the objective is to provide evidence of effectiveness under real world roll-out, monitoring adherence is likely to be less useful at an interim assessment point; any steps to improve compliance might deviate from the pragmatic objective.^28^ Nonetheless, in pragmatic trials, it can still be of interest to document compliance, as part of fidelity assessment, to triangulate and explain subsequent findings, but this is usually undertaken using a process evaluation.^37^ Certain cluster trials, such as those evaluating individual-level therapeutic interventions, might still warrant monitoring for compliance.

## Case study

**Figure 1.**
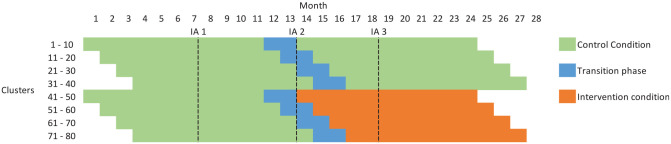
Schematic representation of E-MOTIVE Under anticipated cluster recruitment, assessment 1 (25% point, IA1) will take place roughly at month 7 (clusters will have participated between 3 and 7 months), not outlined here as involves only monitoring completeness of data; assessment 2 (50% point, IA2) will take place roughly at month 13 (clusters will have participated between 9 and 13 months although only observations observed under usual care will be included, that is between 9 and 11 months); and assessment 3 (75% point, IA3) at month 18 (clusters will have participated between 14 and 18 months, including only intervention data).

The E-MOTIVE study is a CRT of an intervention (the E-MOTIVE first-response bundle) designed to treat excessive bleeding after birth. The study is set across four countries and uses a cluster randomised design with a baseline control phase, where clusters are health facilities (Figure 1, Supplementary Table 1). The primary outcome is a composite of postpartum haemorrhage, laparotomy and maternal death. Outcomes will be assessed on all vaginal births, with no exclusions and no individual participant recruitment. Full details of the trial protocol are available elsewhere.^38^ The trial is registered at https://clinicaltrials.gov/ct2/show/NCT04341662.

It is anticipated that 80 health facilities will enter an 11-month baseline period in which they will be following usual care. After this baseline period, 40 of the 80 health facilities will be randomised to the E-MOTIVE intervention for 11 months, following a 2-month transition phase where the intervention is implemented in the clusters. The other 40 health facilities will continue to follow usual care. The parallel design with a baseline period can have increased statistical efficiency over the conventional parallel design, without inducing as much temporal confounding as a stepped-wedge trial.^39^

### Proposed interim monitoring plan

The study has two independent committees – the trial steering committee and the data monitoring committee. The data monitoring committee will function according to the DAMOCLES charter,^40^ and in addition to the trial-specific objectives will also monitor results from external trials. All interim assessments will be conducted in agreement with a pre-specified data monitoring proposal. In the post-intervention phase, all assessments will be presented blind (interventions labelled as A and B) but will be unblinded if a clear need arises. The independent trial steering committee will ultimately decide whether any modifications of the trial are necessary, under guidance from the data monitoring committee.

It is anticipated that the study will have staggered cluster recruitment, with an estimated 16 clusters recruited each month for 5 months (Figure 1). Interim review of the trial progress (Table 1) has the broad objectives to (1) monitor sample size assumptions, (2) monitor for any evidence of selection bias, and (3) monitor safety data including an interim assessment of the primary outcome. Although not discussed specifically, an additional objective is to monitor the completeness of data (i.e. number with missing information on particular data items, including individual-level characteristics and outcomes). Monitoring adherence is not included as part of the interim monitoring plan but rather will form part of a fidelity assessment in a process evaluation that will run alongside the main trial. There are two proposed interim assessment points. The first will occur after approximately 50% of births have occurred and will only include observations collected under the control condition. The second will take place after 75% of births and will include accrued births under both assigned intervention conditions.

Objective 1: Monitor sample size assumptions and advise whether changes to the sample size are required to ensure the study is sufficiently powered (after 50% of births and including only control data)

**Table 1.** Timeline for interim assessments and roles at each assessment point.

Objective	50% of births (including only pre-randomisation data)	75% of births (including only post**-**randomisation data)
Objective 1: Monitor sample size assumptions and advise whether changes to the sample size are required to ensure the study is sufficiently powered	Estimate prevalence of the primary outcome (with 95% CI); within-period intra-cluster correlations (with 95% CI); cluster sizes and variation of cluster sizes. Perform a sample size re-estimation with no information on treatment status, including only observations under the control condition	
Objective 2: Monitor for any evidence of selection bias by evaluating imbalance in the characteristics and numbers of participants recruited under intervention and control condition		Compare individual-level characteristics and number recruited across intervention conditions. For individual-level characteristics, differences will be reported using mean differences or risk differences with 95% confidence intervals. For numbers recruited across intervention conditions, numbers will be compared to the average of the number recruited per cluster-period under the control condition
Objective 3: Monitor safety data and interim assessment of primary outcome		Compare principle safety data (all-cause maternal deaths and intensive care admissions), the primary outcome and its components across intervention conditions, report risk differences and 99.9% confidence intervals

CI: confidence interval.

Full details of the original sample size calculation are included in Supplementary Material 1. There are several nuisance parameters: the prevalence of the primary outcome, the number of births within clusters, variation in number of births across clusters, the within-period intra-cluster correlation, and the cluster auto-correlation. The within-period intra-cluster correlation should represent the degree of correlation within each cluster over an 11-month period. At the first proposed interim assessment point (after 50% of births), clusters will contribute between 9 and 11 months of data, at which point, it will be possible to estimate the within-period intra-cluster correlation. Estimates of cluster-auto correlations (which can be thought of as a measure of how much correlations decay between the first and second time period) require data over two measurement periods (i.e. over 22 months in this context). It is therefore not possible to provide updated estimates of the cluster-auto correlation as these would not be available until the trial has ended. At the point at which the sample size re-estimation will be conducted, no observations will have been collected under the treatment condition and so group-sequential or conditional power methods will not be used. Sample size re-estimation at later time points would not be actionable due to the extensive undertaking of recruiting extra clusters and the need to report within a fixed time frame. Sample size re-estimation therefore concerns four nuisance parameters.

Sample size re-estimation will involve estimating nuisance parameters along with 95% confidence intervals with appropriate allowances for the clustered nature of the trial (see Supplementary Material 2 for full information on how these estimates will be derived), but will not involve estimation of the treatment effect. Anticipated estimates of these confidence intervals are presented in Table 2. The confidence interval width for the prevalence of the binary outcome, only marginally decreases between estimates that might be made after 25% and 50% of births (note after 13% of births, there is still considerable uncertainty). Therefore, there is limited value in using data past the 25% assessment point to estimate the prevalence to inform decision-making. However, while after 25% of births, it will be feasible to provide an accurate estimate of the prevalence of the primary outcome, it will not be possible to estimate within-cluster correlations at this point (Table 3). Without good information on within-cluster correlations, Table 4 reveals that sample size re-estimation will not afford any actionable decisions. Therefore, one planned assessment of sample size is planned at the 50% assessment point. Minimising the number of sample size re-estimations without using information on treatment status is likely to reduce risks of inflating type-1 errors.

**Table 2.** Confidence interval estimates for the prevalence of the primary outcome at different interim assessment points under various assumptions.

Prevalence of primaryoutcome (proportion)	Assessment time(percentage of births)	95% confidence interval for prevalence of primary outcome			
		WP-ICC = 0.001	WP-ICC = 0.01	WP-ICC = 0.02	WP-ICC = 0.05
0.005	13%	0.0042–0.0058	0.0033–0.0067	0.0027–0.0073	0.0015–0.0085
	25%	0.0043–0.0057	0.0033–0.0067	0.0027–0.0073	0.0014–0.0086
	50%	0.0044–0.0056	0.0034–0.0066	0.0028–0.0072	0.0015–0.0085
0.01	13%	0.0088–0.0112	0.0076–0.0124	0.0068–0.0132	0.0050–0.0150
	25%	0.0090–0.0110	0.0076–0.0124	0.0067–0.0133	0.0049–0.0151
	50%	0.0091–0.0109	0.0077–0.0123	0.0068–0.0132	0.0050–0.0150
0.015	13%	0.0136–0.0164	0.0121–0.0179	0.0110–0.0190	0.0089–0.0211
	25%	0.0138–0.0162	0.0121–0.0179	0.0110–0.0190	0.0087–0.0213
	50%	0.0139–0.0161	0.0122–0.0178	0.0111–0.0189	0.0089–0.0211
0.02	13%	0.0183–0.0217	0.0166–0.0234	0.0154–0.0246	0.0130–0.0270
	25%	0.0186–0.0214	0.0166–0.0234	0.0154–0.0246	0.0128–0.0272
	50%	0.0188–0.0212	0.0168–0.0232	0.0155–0.0245	0.0130–0.0270
0.025	13%	0.0232–0.0268	0.0212–0.0288	0.0199–0.0301	0.0172–0.0328
	25%	0.0234–0.0266	0.0212–0.0288	0.0198–0.0302	0.0169–0.0331
	50%	0.0236–0.0264	0.0214–0.0286	0.0200–0.0300	0.0172–0.0328
0.03	13%	0.0377–0.0423	0.0353–0.0447	0.0336–0.0464	0.0302–0.0498
	25%	0.0380–0.0420	0.0353–0.0447	0.0335–0.0465	0.0299–0.0501
	50%	0.0383–0.0417	0.0355–0.0445	0.0338–0.0462	0.0302–0.0498
0.04	13%	0.0377–0.0423	0.0353–0.0447	0.0336–0.0464	0.0302–0.0498
	25%	0.0380–0.0420	0.0353–0.0447	0.0335–0.0465	0.0299–0.0501
	50%	0.0383–0.0417	0.0355–0.0445	0.0338–0.0462	0.0302–0.0498

WP-ICC: within-period intra-cluster correlation.

Anticipated sample size: 46,080 observations from 80 clusters (13% assessment point); and 84,480 observations from 80 clusters (25% assessment point) and 165,120 observations from 80 clusters (50% assessment). For illustration, the table also depicts values at assessment points (when 13% and 25% of observations have been collected), even though data will not be monitored at this point. This assumes there has been no cluster drop out.

**Table 3.** Confidence interval for the within-period intra-cluster correlation after 50% of births.

WP-ICC	0.001	0.01	0.02	0.05
95% confidence interval	0.00051–0.00149	0.00673–0.01327	0.01371–0.02629	0.03501–0.06499

WP-ICC: within-period intra-cluster correlation.

Assumes at the 50% assessment point there are data from 80 clusters and 165,120 observations; This assumes there has been no cluster drop out.

**Table 4.** Comparison of power and the number of clusters required for 90% power under likely scenarios.

Prevalence of primary outcome		WP-ICC = 0.001	WP-ICC = 0.01	WP-ICC = 0.02	WP-ICC = 0.05
0.005	Power (72 clusters)	77.8	51.2	37.9	22.1
	Power (80 clusters)	81.9	55.4	41.3	24.0
	Clusters required	102	192	278	536
0.01	Power (72 clusters)	97.2	80.5	64.8	39.2
	Power (80 clusters)	98.3	84.4	69.4	42.8
	Clusters required	52	96	140	268
0.015	Power (72 clusters)	99.7	93.3	81.9	54.4
	Power (80 clusters)	99.9	95.4	85.7	58.8
	Clusters required	34	64	92	178
0.02	Power (72 clusters)	99.9	98.0	**91.4**	66.8
	Power (80 clusters)	99.9	98.8	**93.9**	71.4
	Clusters required	26	48	**72**	132
0.025	Power (72 clusters)	99.9	99.4	96.1	76.6
	Power (80 clusters)	99.9	99.7	97.5	80.8
	Clusters required	22	38	56	106
0.03	Power (72 clusters)	99.9	99.8	98.3	83.8
	Power (80 clusters)	99.9	99.9	99.1	87.4
	Clusters required	18	32	46	88
0.04	Power (72 clusters)	99.9	99.9	99.7	92.8
	Power (80 clusters)	99.9	99.9	99.9	95.0
	Clusters required	14	24	34	66

WP-ICC: within-period intra-cluster correlation.

Sample size calculations have assumed a cluster auto-correlation of 0.97, 2,112 observations per cluster per period, a coefficient of variation of cluster sizes of 0.5, and are to detect a relative risk reduction of 25%. See supplementary material 1 for full details of power calculation implementation and methods. Base case highlighted in bold represents assumed values of parameters in sample size calculation which obtains 90% power). ‘Clusters required’ represents number of clusters needed to obtain 90% power before allowing for 10% drop out.

However, even at the 50% assessment point, there might be considerable uncertainty of the nuisance parameters. Furthermore, any increase in sample size suggested by the sample size re-estimation might not be feasible. Therefore, the data monitoring committee will be provided with the estimated nuisance parameters, their uncertainty, and estimates of study power (to detect the apriori specified target effect size) under all plausible scenarios. They will be asked to consider if the trial should seek permission from its funder to increase the number of clusters recruited (increasing the cluster size by extending the duration of the study was not thought to be appropriate). It is unlikely that the study will be stopped based on sample size re-estimation as it will not be possible to be certain the trial does not have adequate power to detect larger target differences (Supplementary Table 2).

Objective 2: Monitor for any evidence of selection bias by evaluating imbalance in the characteristics and numbers of participants recruited under intervention and control condition (after 75% of births).

With appropriate ethical approvals, the study does not use individual participant recruitment and also has broad eligibility criteria. This means that the trial should be at moderate to low risk of identification and recruitment biases. Nonetheless, because it is an unblinded evaluation with post-randomisation identification of participants, there is a plan to monitor the trial for selection bias. Selection bias might manifest as either differences in total number of observations across intervention and control conditions or differences in the individual-level characteristics. This assessment will take place after 75% of births, when clusters have accrued births under assigned intervention conditions and will involve the comparison set of pre-specified individual-level characteristics.

To aid this assessment differences in individual-level characteristics between intervention conditions (during the post-intervention phase) along with 95% confidence intervals appropriately allowing for the clustered nature of the design, will be computed. These characteristics are all binary or continuous. For binary characteristics (e.g., previous preterm birth) differences in proportions will be estimated using mixed-effect binomial regression models with identity link. In the case of non-convergence, odds ratios will be estimated from mixed-effects logistic regression. For continuous characteristics (e.g., maternal age), difference in means will be computed using a mixed-effect linear model (with appropriate transformations where residuals are not normally distributed). For numbers of participants accrued the total number of observations and the average cluster size (summarised either using mean and standard deviation or median and inter-quartile range as appropriate) will be determined. To help identify differences in expected numbers of births identified as eligible, the available births per cluster-month will be compared to that expected based on the average numbers per cluster-month in the control period.

The data monitoring committee will be asked to consider whether any differences (between study arms) in total numbers recruited or the individual-level characteristics across intervention conditions are clinically important, and whether there is evidence that the differences are unlikely to be due to chance alone. As part of this, the committee will be asked to reflect on whether if the full trial were to exhibit such imbalances, would this imbalance question the reliability and validity of the results. In the event that selection bias is identified, the panel will be tasked with considering the following mitigation options: (1) investigation of whether all eligible births are being assessed as eligible, and if not, why not; and as a last resort and only in the case of large differences (2) changing pre-specified primary analysis from unadjusted to covariate adjusted. Of note, the primary pre-specified analysis will be adjusted for covariates used in the randomisation and a fully pre-specified covariate adjustment will be included as a sensitivity analysis (using a propensity score approach^23^). Any change to a primary fully adjusted individual-level covariate analysis will include all those covariates in the pre-specified sensitivity analysis and not be based on covariates identified as statistically significant at the interim monitoring. This change would only be made in the very unlikely event there was a consensus that the data were more aligned with an observational study than a randomised evaluation.

Objective 3: Monitor safety data and interim assessment of primary outcome (after 75% of births)

The intervention in the E-MOTIVE trial is unlikely to bring any harm. As a result, it was decided not to continuously monitor any serious adverse events. The 75% assessment point will however include a comparison of principle safety data (all-cause maternal deaths and intensive care admissions), the primary outcome and its components. This information will be presented stratified by intervention condition with a measure of differences (i.e. risk difference) with 99.9% confidence intervals (appropriately adjusted for clustering). Presenting 99.9% confidence intervals is equivalent to the Haybittle–Peto approach of adopting a p value of 0.001. This criterion means there should be minimal concern regarding the inflation of type-I error rates; yet also means that no correction to p values (and subsequent confidence intervals) is required at the main analysis. The data monitoring committee will be advised to consider whether any differences in these key outcome data is clinically important and unlikely to be due to chance. In the unlikely event that differences look unlikely to be due to chance (i.e. the confidence interval mostly supports benefit in the one direction) the committee can ask for the data to be unblinded.

## Conclusion

The interim analyses of cluster trials raise many practical issues. The monitoring might include that which routinely forms part of monitoring in individually randomised trials: harm and outcomes, as well as protocol implementation, data quality, and sample size re-estimation. However, because cluster randomisation is typically used to evaluate low-risk interventions, monitoring for safety is less likely to be required. Moreover, the use of cluster randomisation likely means other factors need to be considered at interim monitoring. For example, at sample size re-estimation, there are typically more nuisance parameters and uncertainty around these can be large. This might mean that increasing or decreasing sample size will not always be appropriate or feasible. Interim monitoring of cluster trials also has the potential to identify selection bias. Finally, the pragmatic nature of cluster trials might mean that the utility of methods to allow for interim monitoring of outcomes based on statistical testing, or monitoring for adherence to study interventions, are less relevant. By presenting the proposed data monitoring plan for the E-MOTIVE trial, our intention is to stimulate debate on these issues, ultimately leading to better practice and possibly recommendations for good practice.

## Supplemental Material

sj-docx-1-ctj-10.1177_17407745211024751 – Supplemental material for Interim data monitoring in cluster randomised trials: Practical issues and a case study Click here for additional data file. Supplemental material, sj-docx-1-ctj-10.1177_17407745211024751 for Interim data monitoring in cluster randomised trials: Practical issues and a case study by K Hemming, J Martin, I Gallos, A Coomarasamy and L Middleton in Clinical Trials

sj-docx-2-ctj-10.1177_17407745211024751 – Supplemental material for Interim data monitoring in cluster randomised trials: Practical issues and a case study Click here for additional data file. Supplemental material, sj-docx-2-ctj-10.1177_17407745211024751 for Interim data monitoring in cluster randomised trials: Practical issues and a case study by K Hemming, J Martin, I Gallos, A Coomarasamy and L Middleton in Clinical Trials
