# Supplementary material for: Interim data monitoring in cluster randomised trials: Practical issues and a case study
Source: Clin Trials. 2021 Jun 22;18(5):552–61. doi: 10.1177/17407745211024751 (PMC8479148; doi:10.1177/17407745211024751)
Supplement: sj-docx-1-ctj-10.1177_17407745211024751 – Supplemental material for Interim data monitoring in cluster randomised trials: Practical issues and a case study [file sj-docx-1-ctj-10.1177_17407745211024751.docx]

**Supplementary Material 1**

*Sample size justification*

The expected number of eligible births per cluster-month is 192. The cluster size per period is 2,112 (=11*192) and the anticipated total cluster size is 4,224 (22*192). The planned total sample size, assuming there are 80 clusters in the trial (running for 22 months), evenly split across the intervention and control groups, is 337,920 (=80*192*22). The number of clusters (K=80) has been inflated by 10% to allow for drop out from the number of clusters required under the base-case sample size estimate (K=72). The methods to justify the sample size in a cluster randomised trial with a baseline period have been described elsewhere^[[1]](#endnote-1)^ and implemented in an RShiny App (https://clusterrcts.shinyapps.io/rshinyapp/). Full details on the values used in these calculations, justification for these choices, and an exploration of sensitivity, are provide below.

This sample size is expected to be sufficient to provide 90% power at 5% significance to detect a change from 2% to 1.5% (25% relative reduction) in the primary outcome, after allowing for clustering (assuming a within-period ICC of 0.02 and cluster-auto correlation of 0.97), for varying cluster sizes (assuming a coefficient of variation of 0.5), and allowing for 10% of clusters to drop-out.

The expected prevalence of the primary composite outcome was estimated to be 2%, estimated from the CHAMPION trial^[[2]](#endnote-2)^ and we considered sensitivity across the range 1.5% to 4%. A relative risk reduction of 25% was considered to be a clinically meaningful difference to detect, though smaller reductions could also be worthwhile. Under some scenarios this target effect size might not be detectable at 90% power, and so we have thus considered sensitivity across the range of relative reduction of 20% to 30%.

Sample size calculations have allowed for the clustered nature of the design. As recommended, values for the intra cluster correlations have been informed through a combination of the literature and analysis of available data on a similar set of outcomes. As the trial is run over two periods we require an estimate of the within-period intra cluster correlation. Analysis of the CHAMPION trial estimated the within-period intra-cluster correlation to be 0.03 (95% CI: 0.02 to 0.05) for a similar composite outcome of postpartum haemorrhage ≥1000 ml, laparotomy and maternal death. This was estimated using a linear mixed regression model and so is on the proportions scale as is appropriate for a sample size calculation. As the literature suggests, rare clinical outcomes tend to have small intra-cluster correlations^[[3]](#endnote-3)^, for this reason we have considered an extended range from 0.001 to 0.05.

To allow for variations in clustering over time, we have allowed for a cluster by period random effect. This has been incorporated in the sample size calculations using the cluster auto-correlation. There is limited information from the literature on likely values of the cluster auto-correlation. Using data from the CHAMPION trial, we created two 11-month periods, which match the planned study design. From this, we estimated a cluster auto-correlation of 0.97, and as no current methodology exists to calculate a confidence interval for this value, we considered sensitivity to the range 0.95 to 1.0.

Survey data from health facilities that will be included in the trial was collected to obtain information on the number of births per facility. From this survey data, we calculated an average number of births per health facility per month of 192. The health facilities varied in size. We have estimated a coefficient of variation of cluster sizes of 0.5 and have allowed for this in our sample size calculations using a conservative correction.

**Supplementary Material 2**

*Confidence interval for the prevalence of the primary outcome*

In a cluster randomised trial, the sample size is inflated over that required for an individually randomised trial by a design effect [1+(m-1)$\rho$], where *m* is the cluster size and $\rho$ is the intra-cluster correlation. In our situation the intra-cluster correlation represents the clustering within any given time period and so is replaced by the within-period intra-cluster correlation. When cluster sizes vary this design-effect is further modified^[[4]](#endnote-4)^:

$$DE=\frac{1+\left( m-1 \right)\rho}{1-\left( {cv}^{2} \times\left( \frac{m\rho}{m\rho+1-\rho}\left( 1- \frac{m\rho}{m\rho+1-\rho} \right) \right) \right)}$$

where *cv* is the coefficient of the variation in cluster sizes (anticipated to be 0.5 in this study). We use this design effect to inflate the confidence interval width for the estimated prevalence of the primary outcome^[[5]](#endnote-5)^. The standard error of the estimated proportion, as estimated at any given point in the interim assessments, is thus:

$${SE}(p)=\frac{p\left( 1-p \right)}{N}\times DE$$

Where *SE(p)* is the standard error for the prevalence of the primary outcome (adjusted for clustering), *p* is the proportion with the primary outcome, and *N* is the sample size.

We have used this approach to estimate the confidence intervals for the expected prevalence (2%) at each of our interim analysis points, using our expected within-period intra-cluster correlation (0.02) and assuming normality. We have considered sensitivity by examining the range of prevalence values from 0.5%, 1%, 1.5%, 2.5%, and 3%. To consider sensitivity to the within-period intra-cluster correlation we allowed this to vary from 0.001, 0.01, and 0.05. We use normal approximations for calculation of confidence intervals, and with 80 clusters this is likely to be a reasonable approximation.

*Confidence interval for the within-period intra-cluster correlation*

The confidence interval for the within-period intra-cluster correlation is approximated using the delta method ^[[6]](#endnote-6)^. To this end, the variance is:

$$Var\left( \rho\right)=\frac{2\left( 1-\rho\right)^{2}\left( DE \right)^{2}}{m\left( m-1 \right)\left( K-1 \right)}$$

Where *m* is the cluster size, *K* is the total number of clusters, and *DE* is a design effect to allow for varying cluster size (see above). This variance can be used to calculate a confidence interval, assuming normality. Using the expected number of participants at 11 months (2,112 observations in each of 80 clusters) and the expected WP-ICC (0.02), we have estimated the confidence interval for this value. We have considered sensitivity to this by considered a range of WP-ICC values (0.001, 0.01, and 0.05).

*Estimation of power*

For each of the likely scenarios described above, we have estimated the power under the given design, using methods proposed by Hooper and Bourke^[[7]](#endnote-7)^ implemented in an R Shiny app^1^. For all scenarios, we have assumed a cluster auto-correlation of 0.97 (based on CHAMPION trial), 2,112 participants per cluster per period (under a two-period design), and a coefficient of variation (of cluster sizes) of 0.5 (based on survey data, but to be updates in light of trial data). For each scenario, we have also estimated the number of clusters that would be required to obtain 90% power.

**References**

1. Hemming K, Kasza J, Hooper R, Forbes A, Taljaard M. A tutorial on sample size calculation for multiple-period cluster randomized parallel, cross-over and stepped-wedge trials using the Shiny CRT Calculator. International Journal of Epidemiology. 2020 Feb 22. [↑](#endnote-ref-1)
2. WOMAN Trial Collaborators. Effect of early tranexamic acid administration on mortality, hysterectomy, and other morbidities in women with post-partum haemorrhage (WOMAN): an international, randomised, double-blind, placebo-controlled trial [published correction appears in Lancet. 2017 May 27;389(10084):2104]. Lancet. 2017;389(10084):2105-2116. doi:10.1016/S0140-6736(17)30638-4 [↑](#endnote-ref-2)
3. Gulliford MC, Adams G, Ukoumunne OC, Latinovic R, Chinn S, Campbell MJ. Intraclass correlation coefficient and outcome prevalence are associated in clustered binary data. J Clin Epidemiol. 2005;58(3):246-251. doi:10.1016/j.jclinepi.2004.08.012 [↑](#endnote-ref-3)
4. van Breukelen GJ, Candel MJ, Berger MP. Relative efficiency of unequal versus equal cluster sizes in cluster randomized and multicentre trials. Statistics in medicine. 2007 Jun 15;26(13):2589-603. [↑](#endnote-ref-4)
5. Franco C, Little RJ, Louis TA, Slud EV. Comparative Study of Confidence Intervals for Proportions in Complex Sample Surveys. Journal of survey statistics and methodology. 2019 Sep 1;7(3):334-64. [↑](#endnote-ref-5)
6. Shoukri MM, Donner A, El-Dali A. Covariate-adjusted confidence interval for the intraclass correlation coefficient. Contemporary clinical trials. 2013 Sep 1;36(1):244-53. [↑](#endnote-ref-6)
7. Hooper R, Bourke L. Cluster randomised trials with repeated cross sections: alternatives to parallel group designs. BMJ. 2015;350:h2925. Published 2015 Jun 8. doi:10.1136/bmj.h2925 [↑](#endnote-ref-7)
