# Supplementary material for: Interim data monitoring in cluster randomised trials: Practical issues and a case study
Source: Clin Trials. 2021 Jun 22;18(5):552–61. doi: 10.1177/17407745211024751 (PMC8479148; doi:10.1177/17407745211024751)
Supplement: sj-docx-2-ctj-10.1177_17407745211024751 – Supplemental material for Interim data monitoring in cluster randomised trials: Practical issues and a case study [file sj-docx-2-ctj-10.1177_17407745211024751.docx]

**Supplementary Table 1: Expected data available at different interim assessment points**

| **Interim assessment point** | | | | | | |
| --- | --- | --- | --- | --- | --- | --- |
|  | **25% of births (including only pre-randomisation data)** | | **50% of births (including only pre-randomisation data)** | | **75% of births (including only post randomisation data)** | |
| Study month | 7 | | 13 | | 18 | |
| Months of data available per cluster | 4 to 7 | | 10 to 11 | | 13 to 16 | |
| Number of months and number of clusters available | 4 | 20 | 10 | 20 | 13 | 20 |
|  | 5 | 20 | 11 | 60 | 14 | 20 |
|  | 6 | 20 |  |  | 15 | 20 |
|  | 7 | 20 |  |  | 16 | 20 |
| Total months of available data  (Sum of number of clusters x number of months) | 440  (= 4x20 + 5x20 + 6x20 + 7x20) | | 860  (= 10x20 + 11x60) | | 1,160  (= 13x20 + 14x20 + 15x20 + 16x20) | |
| Total available sample size | 84,480  (= 440 x 192) | | 165,120  (= 860 x 192) | | 222,720  (= 1,160 x 192) | |

**Supplementary Table 2: Estimates of the power available under different scenarios for key design parameters*.***

|  | | | RRR = 20% | | | | | | | RRR = 25% | | | | | | | RRR = 30% | | | | | | |
| --- | --- | --- | --- | --- | --- | --- | --- | --- | --- | --- | --- | --- | --- | --- | --- | --- | --- | --- | --- | --- | --- | --- | --- |
|  |  |  | Control prevalence of the primary outcome | | | | | | | | | | | | | | | | | | | | |
|  |  |  | 0.5% | 1% | 1.5% | 2% | 2.5% | 3% | 4% | 0.5% | 1% | 1.5% | 2% | 2.5% | 3% | 4% | 0.5% | 1% | 1.5% | 2% | 2.5% | 3% | 4% |
| CAC = 0.95 | WP-ICC | 0.001 | 55.9 | 84.8 | 96.4 | 98.9 | 99.7 | 99.9 | 99.9 | 76.2 | 96.6 | 99.6 | 99.9 | 99.9 | 99.9 | 99.9 | 90.2 | 99.6 | 99.9 | 99.9 | 99.9 | 99.9 | 99.9 |
|  |  | 0.01 | 28.3 | 50.1 | 67.4 | 79.6 | 87.8 | 92.9 | 97.8 | 41.9 | 70.1 | 86.3 | 94.2 | 97.7 | 99.2 | 99.9 | 57 | 85.8 | 96.1 | 94.2 | 99.8 | 99.9 | 99.9 |
|  |  | 0.02 | 19.5 | 34.5 | 54.6 | 60.2 | 70 | 77.9 | 92.9 | 28.6 | 50.8 | 68.1 | 80.3 | 88.3 | 93.3 | 98 | 39.7 | 67.3 | 84.1 | 92.8 | 97 | 98.8 | 99.8 |
|  |  | 0.05 | 11.5 | 18.9 | 30.8 | 33.4 | 40.4 | 47 | 67.3 | 16 | 27.6 | 38.9 | 49.3 | 58.5 | 66.6 | 79.1 | 21.6 | 38.4 | 53.3 | 65.7 | 75.4 | 82.8 | 92.1 |
| CAC = 0.97 |  | 0.001 | 57.5 | 86.1 | 96.8 | 99.3 | 99.8 | 99.9 | 99.9 | 77.7 | 97.1 | 99.7 | 99.9 | 99.9 | 99.9 | 99.9 | 91.3 | 99.7 | 99.9 | 99.9 | 99.9 | 99.9 | 99.9 |
|  |  | 0.01 | 34.8 | 60.4 | 77.9 | 88.5 | 94.3 | 97.3 | 99.5 | 51.1 | 80.5 | 93.3 | 98 | 99.4 | 99.8 | 99.9 | 67.8 | 92.9 | 98.8 | 98 | 99.9 | 99.9 | 99.9 |
|  |  | 0.02 | 25.5 | 45.4 | 67.8 | 80.1 | 83.6 | 89.7 | 97.9 | 37.9 | 64.8 | 81.9 | 91.4 | 96.1 | 98.3 | 99.7 | 52 | 81.3 | 93.8 | 98.2 | 99.5 | 99.9 | 99.9 |
|  |  | 0.05 | 15.3 | 26.4 | 43 | 54.2 | 56.3 | 64.3 | 83.8 | 22.1 | 39.2 | 54.4 | 66.8 | 76.5 | 83.8 | 92.8 | 30.4 | 53.7 | 71.2 | 83 | 90.4 | 94.8 | 98.6 |
| CAC = 1.0 |  | 0.001 | 60.2 | 88.2 | 97.4 | 99.9 | 99.9 | 99.9 | 99.9 | 80.3 | 97.9 | 99.9 | 99.9 | 99.9 | 99.9 | 99.9 | 92.8 | 99.8 | 99.9 | 99.9 | 99.9 | 99.9 | 99.9 |
|  |  | 0.01 | 55.2 | 84.2 | 95.3 | 98.8 | 99.7 | 99.9 | 99.9 | 76.6 | 96.4 | 99.6 | 99.9 | 99.9 | 99.9 | 99.9 | 89.7 | 99.5 | 99.9 | 99.9 | 99.9 | 99.9 | 99.9 |
|  |  | 0.02 | 55.1 | 84.2 | 95.8 | 98.9 | 99.7 | 99.9 | 99.9 | 75.5 | 96.4 | 99.7 | 99.9 | 99.9 | 99.9 | 99.9 | 89.7 | 99.6 | 99.9 | 99.9 | 99.9 | 99.9 | 99.9 |
|  |  | 0.05 | 56.2 | 85.1 | 95.4 | 98.8 | 99.8 | 99.9 | 99.9 | 76.5 | 96.7 | 99.9 | 99.9 | 99.9 | 99.9 | 99.9 | 90.5 | 99.6 | 99.9 | 99.9 | 99.9 | 99.9 | 99.9 |
| Note: The cell colour indicates the power: red (<70%); yellow (70% to 80%); light green (80% to 90%); and dark green (>90%). Power has been calculated assuming 72 health facilities, with an average number of 192 births per month (22-month study), and a coefficient of variation of cluster sizes of 0.5. CAC: Cluster Auto-Correlation; WP-ICC: within-period intra cluster correlation. RRR = Relative Risk Reduction; base case highlighted in bold represents assumed values of parameters in sample size calculation which obtains 90% power). | | | | | | | | | | | | | | | | | | | | | | | |
